# Osteoglycin (OGN) reverses epithelial to mesenchymal transition and invasiveness in colorectal cancer via EGFR/Akt pathway

**DOI:** 10.1186/s13046-018-0718-2

**Published:** 2018-03-02

**Authors:** Xiang Hu, Ya-Qi Li, Qing-Guo Li, Yan-Lei Ma, Jun-Jie Peng, San-Jun Cai

**Affiliations:** 1 Department of Colorectal Surgery, Fudan University Shanghai Cancer Center, 270 Dong’an Road, Shanghai, 20032 China; 2 Department of Oncology, Shanghai Medical College, Fudan University, Shanghai, 200032 China

**Keywords:** Osteoglycin, EGFR, Zeb-1, EMT

## Abstract

**Background:**

Many types of cancers are devoid of the small leucine-rich proteoglycans: osteoglycin (OGN), but its role in tumorigenesis is poorly studied especially in colorectal cancers (CRC). Here we aim to evaluate the relationship between OGN expression patterns and the clinical course of CRC, and the role of OGN in cancer progression.

**Methods:**

The tissue microarray staining was performed and the relevance between OGN expression and oncologic outcomes was performed using Cox regression analysis. The effect of OGN on cell proliferation and tumorigenesis was examined in vitro and in vivo. Immunoprecipitation assay, immunofluorescence analysis and internalization assay were used for mechanistic study.

**Results:**

Patients with high expression of OGN were associated with a profound longer survival in CRC and the high serum OGN level was also indicative of fewer recurrences consistently. In colon cancer cells, OGN increased dimerization of EGFR, then triggered EGFR endocytosis and induced the recruitment of downstream components of the EGFR internalization machinery (Eps15 and epsin1). Above all, OGN reduced Zeb-1 expression via EGFR/Akt leading to inhibition of epithelial-mesenchymal transition. As results, in vitro and in vivo, the OGN expression was demonstrated to reduce cell proliferation, inhibit invasion of colon cancer cells then impede cancer progression.

**Conclusions:**

There is a positive association between OGN level and prolonged survival in CRC. OGN plays a restrictive role in colorectal cancer progression by reduced activation of EGFR/AKT/Zeb-1.

## Background

Small leucine-rich proteoglycans (SLRPs) are proteoglycans secreted by a wide range of cells, and therefore are involved in many processes, such as protein–protein interactions, signal transduction, cell adhesion, and DNA repair [1]. SLRP family are extensively studied for both their ability of binding collagen and the ability of performing outside-in signaling [2]. Because of the diversity in their leucine-rich repeats cores and glycosylation patterns, SLRPs can bind several growth factors such as: TGF-β [3], cell surface receptors: epidermal growth factor receptors [4] and insulin growth factor receptors [4]. OGN, one of these SLRPs proteins, is our focus, as it is the least understood. Decreased OGN expression was observed in a variety of different cancers including gastric cancer [5], colorectal adenoma [6], squamous cervical and vaginal cancer [7], invasive ductal breast carcinoma [8], laryngeal carcinoma [9], in comparison with control normal tissues. Even more, OGN expression in normal tissue, benign follicular adenoma progressing to aggressive follicular cancer experienced a progressive decrease, indicating that the level of OGN expression paralleled thyroid tumor progression [10]. Additionally, when OGN was over-expressed in mouse hepatocarcinoma cells by extrinsic transfection, decreased migration and invasion capacity as well as decreased metastasis to peripheral lymph nodes were displayed [11]. Despite several studies demonstrating consistent OGN decrease in cancers, functional data on how OGN is involved in cancer pathology, especially colorectal cancers are lacking, and further research is needed.

EGFRs are frequently activated and cross-talked with other pathophysiology, such as epithelial-mesenchymal transition (EMT), carcinoma angiogenesis in human CRC and play important roles in tumor development and progression [12]. In the tumorigeneses, it fails to negatively feedback to prevent the dire consequences of uncontrolled activation of EGFR. At the meantime, other members of SLRPs were found to bind EGFR resulting in EGFR degradation and attenuation of its downstream signal [13]. And OGN previously was indicated to inhibit cell proliferation, which is rather general and not cell type specific. Based on these previous findings, we will measure oncologic outcomes for CRC based upon OGN expression patterns, further test the effect of OGN on EGFR signaling.

## Methods

### Antibodies and reagents

The following antibodies and reagents were used: anti-OGN (Western blot) from R&D Systems anti-human OGN (IHC) from Sigma-Aldrich; anti-Akt, anti-phospho-Akt (Ser473), anti-phospho-EGFR (Y1068), anti-EGFR, anti-Slug, anti-Erk1/2, anti-Zeb-1, anti-Snail, anti-Twist, anti-Snail from Cell Signaling Technology; anti-CD31, anti-β-actin from prteintech; cross-linking reagent BS3 from Thermo Scientific Pierce; Eps15, epsin1 from Santa Cruz Biotechnology; AKT activator: sc79 from Selleck; OGN human enzyme-linked immunosorbent assay (ELISA) kit from USCN. All chemicals, unless otherwise specified, were purchased from Sigma-Aldrich.

### Study population

The protocol of current study was reviewed and approved by the specialty committee on ethics of biomedicine research, Fudan University Shanghai Cancer Center (FUSCC). The tissue acquisition and utilization followed the National Regulations on the Use of clinical samples in China. All patients from FUSCC dataset have provided written informed consent. The enrollment criteria included: (1) pathologically confirmed with primary colorectal adenocarcinoma and no history of other cancers; (2) undergoing curative surgical resection, but without any preoperative anticancer treatment; and (3) with complete clinical and follow-up data.

### Tissue microarray(TMA) construction and immunohistochemistry(IHC) staining

The TMA used for this study includes unselected, non-consecutive, primary, and sporadic colorectal cancers enrolled between January 2007 and November 2009 in FUSCC. Construction of this TMA has been previously described in detail [14]. Every section was scored independently by two pathologists utilizing a semiquantitative scoring system [15, 16]. The staining intensity was scored as 0 (negative), 1 (weak), 2 (medium) or 3 (strong). Extent of staining was scored as 0 (< 5%), 1(5–25%), 2 (26–50%), 3 (51–75%) and 4 (> 75%) according to the percentages of the positive staining areas in relation to the whole carcinoma area. We multiplied the percentage score by the staining intensity score to generate the immunoreactivity score (IRS). High expression of OGN was defined as detectable immunoreactions in cytoplasm and stoma with IRS > 4.

### Cell culture

The human colon cancer cell lines (SW620, HT29, RKO and HCT116), used for cell experiments, originally purchased from the American Type Culture Collection (Manassas, VA), and cultured in medium according to The Defense Technical Information Center recommendation supplemented with 10% FBS (Gibico, Life Technology, Austria), 1% penicillin/streptomycin in a humidified 5% (*v*/v) atmosphere of CO2 at 37 °C.

### Plasmids construction and viral transduction

Human OGN, Zeb-1, Slug, Snail cDNA was chemically synthesized and cloned into a lentiviral expression vector, pCDH-CMV-MCSEF1-Puro generating pCDH-OGN. The human colon cancer cell lines (SW620, HT29, RKO and HCT116) were transfected with the pCDH-OGN expression vector or the control vector.

### Immunofluorescence

Cells were implanted onto a chamber slide for 24 h reaching 80% confluent, then fixed with paraformaldehyde for 30 min, and followed by permeabilized with 0.5% Triton X-100 for 10 min at room temperature, thereafter the primary antibodies for EGFR were added incubation for overnight at 4 °C, and then the alexa flours 488 Goat anti-rabbit (1:200, Invitrogen) was used as the secondary antibody for an hour at room temperature. At last, nuclei were stained with DAPI for 5 min when necessary. Fluorescence images were photographed with a fluorescence microscope.

### Western blot

RIPA lysis buffer was used for protein extraction. Protein from either cell lysates were separated by SDS-PAGE (6–20% gel) and then transferred to PVDF membranes. Membranes were probed with primary antibodies. Following incubation with horseradish peroxidase-conjugated secondary species-specific antibodies, immunoreactive proteins were detected using ECL (Pierce, Thermo Scientific) and detected using a BioImaging System.

### Cell viability, Transwell migration, invasion assays and wound healing assays

Cell viability: A count of 5 × 10^3^ cells/well was plated into 96-well plates in triplicate and were allowed to adhere overnight. After adherence, 10 μl/100 μl of CCK8 solution was added to each well to co-incubate for 2 h at 37 °C. Then, cell viability was measured by spectrophotometrically at 450 nm, and this was recorded as day 1. After 2, 3, 4, 5 and 6 days, cell viability and proliferation were re-assessed.

#### Wound healing assays

A confluent monolayer of cells was cultured overnight and a scratch was introduced with a pipette tip and images of cell migration into the wound were captured at 0, 24 and 48 h using a light microscope. The results are expressed as follows: covered area (inch^2^).

#### Invasion assay

A density of 5 × 10^4^ cells/well was placed into the upper serum-free medium well of 24-well Transwell inserts coated with Matrigel. Cells were allowed to migrate for 48 h challenged by 10% FBS in the lower well. Following incubation, migrated cells on the lower side were fixed and stained using 0.5% crystal violet and quantified by counting cells from 5 captured images per well.

### Xenotransplant murine models

SW620 cells (10^6^ cells/mouse) which transfected with pCDH-OGN or empty vector control were suspended in 100 μL Matrigel, injected subcutaneously into the right flank of nude mice (*n* = 8, male; 4 weeks old Balb/C athymic nude mouse), and allowed to grow for 4 weeks. Tumor growth was monitored using calipers every 5 days and animals were euthanized when tumors reached 10% of body weight. Primary tumors and organs were harvested and fixed in 10% formalin and paraffin embedded for pathological analysis. Tumor volumes were calculated using the following equation: Volume = (d^2^ × D)/2, where D is the long side and d is the short side. For the metastasis assay, stable OGN-SW620 cells (5 × 10^5^ cells) were orthotopically implanted into the spleens of nude mice.

### Statistical analysis

Statistical analysis was performed with SPSS 22.0 (SPSS Inc., Chicago, IL, USA) and GraphPad Prism v.6 (La Jolla, CA, USA). The optimal cut-off values for serum OGN levels were identified by X-tile 3.6.1 software [17] (Yale University, New Haven, CT, USA). Chi-square test was used to analyze the relationship between clinicopathological parameters and OGN expressions. Survival analysis was performed using the Kaplan-Meier method and Cox regression model. For normal or approximately normal variables, Student’s t-test (for two group comparisons) or analysis of variance (three or more groups) was utilized. *P* < 0.05 was considered statistically significant. All confidence intervals (CIs) were stated at the 95% confidence level.

## Results

### Protein expression and Clinicopathological characteristics

OGN is expressed within the epithelial cancer cell and stromal compartments of human colorectal tumors. OGN staining in the epithelium displayed a cytoplasm-accentuated expression, often combined with a clear membrane staining. Staining in the epithelial cells was frequently accompanied with staining in the stroma, while stromal staining was usually diffuse. To evaluate the expression patterns and clinical importance of OGN, we divided patients into two groups with low expression (Low, IRS ≤ 4) and high expression (High, IRS > 4). In 109 CRC patients (40.1%), a high expression of OGN was observed and OGN expression was designated as low in 163 cases (59.9%). No demographic or baseline clinical data were statistically associated with any pattern of OGN expression. Gender, age, histological type, TNM stage, pathological grading, venous/perineural invasion, MS/MMR status, and adjuvant therapy in FUSCC cohorts are shown in Table 1.

**Table 1:** Description of the study population between colorectal cancer patients with osteoglycin low and high expression

Variables, N (%)	OGN		*P* value
	Low(*N* = 163)	High(*N* = 109)	
Gender			
Male	101(62)	62(56.9)	0.40
Female	62(38)	47(43.1)	
Age, years	56.9 ± 10.7	57.3 ± 11.6	0.79
TNM stage			0.43
I	14(8.6)	7(6.4)	
II	49(30.1)	32(29.4)	
III	73(44.8)	58(53.2)	
IV	27(16.6)	12(11.0)	
N stage			0.16
N0	74(45.4)	46(42.2)	
N1	53(32.5)	28(25.7)	
N2	36(22.1)	35(32.1)	
M stage			0.43
M0	136(83.4)	97(89.0)	
M1	27(16.6)	12(11.0)	
Grade			0.55
Well/ moderate	118(72.4)	85(78.0)	
poor	34(20.9)	19(17.4)	
T stage			0.74
T2	27(16.6)	16(14.7)	
T3	30(18.4)	24(22.0)	
T4	106(65.0)	69(63.3)	
Histological type			0.28
Adenocarcinoma	152(93.3)	105(96.3)	
Mucinous	11(6.7)	4(3.7)	
Lymph node examined			0.17
Median	15 ± 7	16 ± 6	
Perineural invasion			0.26
Negative	133(82.1)	95(87.2)	
Positive	29(17.9)	14(12.8)	
Vascular invasion			0.25
Negative	108(66.3)	75(68.8)	
Positive	51(31.3)	34(31.2)	
Adjuvant Chemotherapy			0.37
No	30(18.4)	18(16.5)	
Yes	108(66.3)	80(73.4)	
MS status/MMR status			0.06
MSS/MMR-proficient	111(68.1)	62(56.9)	
MSI/MMR-deficient	52(31.9)	47(43.1)	

*MMR* indicates mismatch repair, *MS* microsatellite, *MSS* microsatellite stability, *MSI* microsatellite instability

### Increased OGN expression predicts better survival in CRC

Cox regression analysis was performed to test the associations between OGN expression and oncologic outcomes, and the univariate Cox regression model implied that OGN expressions, TNM stages, venous/ perineural invasion and adjuvant therapy were associated with prognosis of CRC patients in terms of cancer specific survival (*P* < 0.05, Table 2). Consistently, multivariate analysis after adjustment revealed that OGN expression was also independent prognostic factor for cancer specific survival in CRC patients, besides T stages and adjuvant therapy (*P* < 0.05). Hence, Kaplan-Meier analysis showed the OGN expression was markedly associated with longer time post-surgical resection, with prolonged cancer specific survival as 75.7 months in the High OGN expression group versus 61.6 months in the Low OGN expression group. Moreover, this survival benefit can translate into prolonged disease-free survival (DFS) evidenced by univariate Cox proportional hazards model (Table 3). What is more, in the multivariate Cox proportional hazards model after adjusting for other effects, OGN expression (*p* = 0.035), TNM stages (*p* < 0.001), perineural invasion (*p* = 0.042), and lymph node examined (*p* = 0.032) were all independently associated with improved DFS. Meanwhile, Kaplan-Meier survival analysis for OGN expression also revealed that high OGN expression correlated with a longer disease-free survival (*p* = 0.037).

**Table 2:** Univariate and Multivariate analyses of prognostic factors for cancer specific survival

Variables	Univariate Analysis			Multivariate Analysis		
	Hazard ratio	95% CI	*P* value	Hazard ratio	95% CI	*P* value
OGN						
Low	1 (reference)			1 (reference)		
High	0.609	0.378–0.980	0.041	0.523	0.317–0.862	0.011
Gender				NI		
Male	1 (reference)					
Female	0.816	0.514–1.296	0.389			
Age, y	1.017	0.997–1.038	0.103	NI		
TNM stage						
IV	1 (reference)			1 (reference)		
III	0.227	0.141–0.367	< 0.01	–	–	
II	0.063	0.029–0.138	< 0.01	0.186	0.075–0.461	< 0.001
I	–	–	0.957			0.969
Grade				NI		
Well/ moderate	1 (reference)					
poor	1.265	0.805–1.987	0.308			
T stage						
T2	1 (reference)			1 (reference)		
T3	3.927	0.860–17.924	0.077	4.247	0.910-19.829	0.066
T4	9.419	2.306–38.469	0.002	4.258	1.022–17.744	0.047
N stage						
N0	1 (reference)			1 (reference)		
N1	2.635	1.456–4.768	0.001	0.510	0.234–1.112	0.09
N2	3.713	2.079–6.633	< 0.001	0.595	0.275–1.286	0.186
M stage						
M0	1 (reference)			1 (reference)		
M1	6.886	4.331–10.95	< 0.001	1.314	0.522–3.306	0.562
Lymph node examined	0.963	0.925–1.002	0.065	NI		
Histological type				NI		
Adenocarcinoma	1 (reference)					
Mucinous	0.842	0.308-2.304	0.738			
Perineural invasion						
Negative	1 (reference)			1 (reference)		
Positive	1.798	1.061–3.045	0.029	1.305	0.750–2.272	0.346
Vascular invasion						
Positive	1 (reference)			1 (reference)		
Negative	0.443	0.283–0.692	< 0.01	1.058	0.640-1.750	0.827
Adjuvant CT						
No	1 (reference)			1 (reference)		
Yes	6.366	3.914–10.356	< 0.01	1.945	1.228–3.080	0.005
MS status/MMR status				NI		
MSS/MMR-proficient	1 (reference)					
MSI/MMR-deficient	0.946	0.599–1.493	0.811			

*MMR* indicates mismatch repair, *MS* microsatellite, *MSS* microsatellite stability, *MSI* microsatellite instability

**Table 3:** Univariate and Multivariate analyses of prognostic factors for disease free survival

Variables	Univariate Analysis			Multivariate Analysis		
	Hazard ratio	95% CI	*P* value	Hazard ratio	95% CI	*P* value
OGN						
Low	1 (reference)			1 (reference)		
High	0.651	0.433–.978	0.039	0.627	0.406–0.968	0.035
Gender				NI		
Male	1 (reference)					
Female	0.889	0.597–1.326	0.565			
Age, y	1.014	0.996–1.032	0.125	NI		
TNM stage						
IV	1 (reference)			1 (reference)		
III	0.216	0.140–0.336	< 0.01	–	–	
II	0.071	0.037–0.136	< 0.01	0.215	0.091–0.510	< 0.001
I	0.021	0.003–0.157	< 0.01	0.162	0.017–1.542	0.113
Grade				NI		
Well/ moderate	1 (reference)					
poor	1.067	0.722–1.575	0.745			
T stage						
T2	1 (reference)			1 (reference)		
T3	2.790	1.029–7.564	0.044	3.753	1.228–11.471	0.02
T4	4.854	1.967–11.979	0.001	3.139	1.127–8.744	0.029
N stage						
N0	1 (reference)			1 (reference)		
N1	2.608	1.582–4.298	< 0.001	0.689	0.343–1.383	0.294
N2	3.328	2.026–5.466	< 0.001	0.701	0.348–1.412	0.32
M stage						
M0	1 (reference)			1 (reference)		
M1	6.907	4.517–10.561	< 0.001	3.071	1.316–7.168	0.009
Lymph node examined	0.954	0.922–0.988	0.009	0.961	0.926–0.997	0.032
Histological type				NI		
Adenocarcinoma	1 (reference)					
Mucinous	0.805	0.328-1.977	0.636			
Perineural invasion						
Negative	1 (reference)			1 (reference)		
Positive	2.002	1.276–3.144	0.003	1.646	1.018–2.660	0.042
Vascular invasion						
Negative	1 (reference)			1 (reference)		
Positive	1.841	1.261–2.690	< 0.002	0.911	0.589–1.410	0.676
Adjuvant CT						
No	1 (reference)			1 (reference)		
Yes	1.950	1.524–2.495	< 0.01	1.289	0.851–1.953	0.231
MS status/MMR status				NI		
MSS/MMR-proficient	1 (reference)					
MSI/MMR-deficient	1.060	0.711–1.581	0.774			

*MMR* indicates mismatch repair, *MS* microsatellite, *MSS* microsatellite stability, *MSI* microsatellite instability

**Fig. 1:**
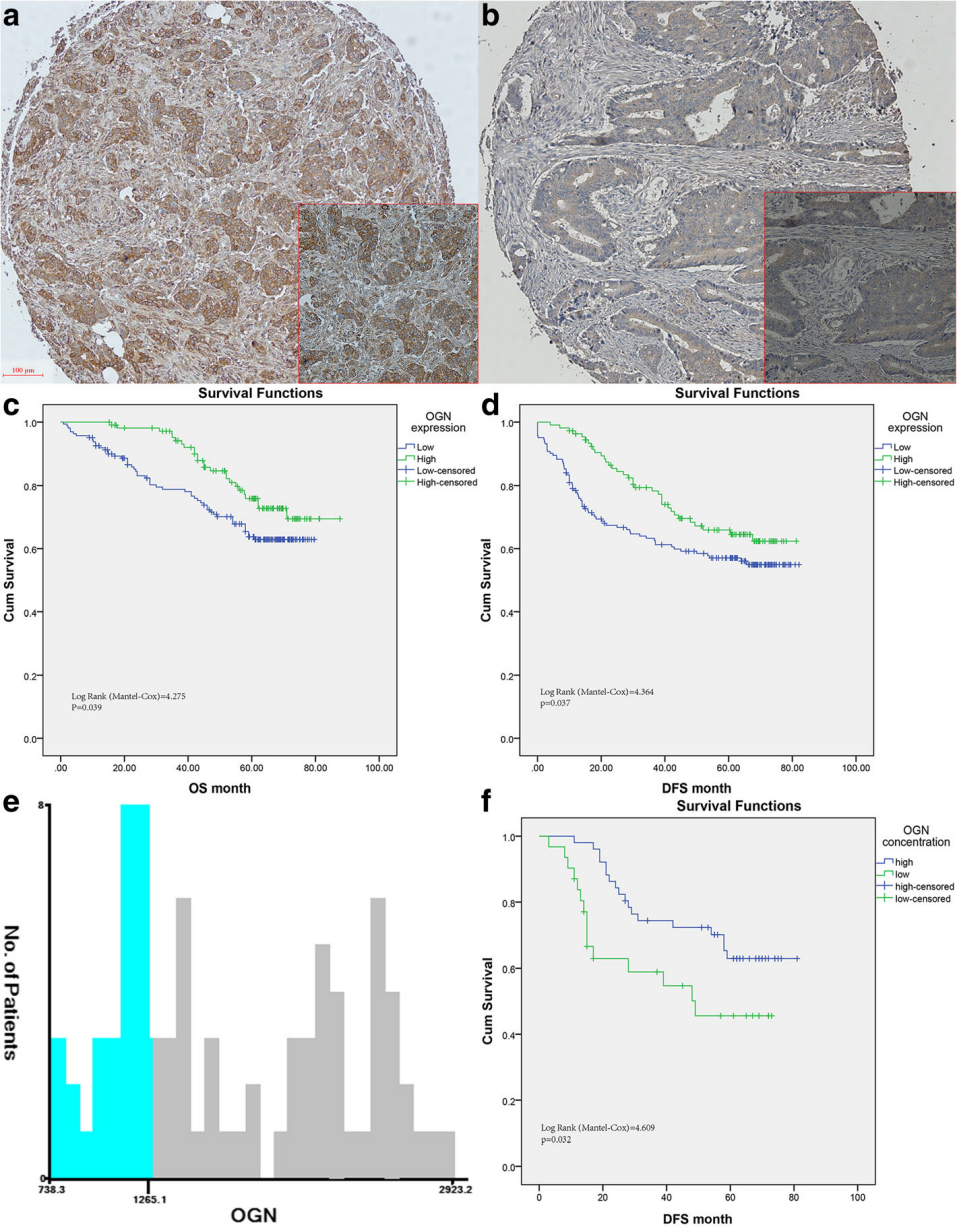
Characteristic expression pattern of OGN in human colorectal cancers. **a** High immunohistochemical staining of OGN in the cytoplasm of cancer cells in TMA samples. The representative pictures of low expression of OGN were also shown (**b**). **c** Kaplan Meier curves for overall survival for High OGN expression versus Low expression group. **d** Disease-free survival for High OGN expression versus Low expression group. **e** The optimal cut-off values of serum OGN levels are shown in histograms of the entire cohort (12.65 ng/ml). **f** Disease-free survival for high serum OGN level versus low serum level group

The potential prognostic effect of OGN expression in CRC patients was further examined by the level of serum OGN. In the FUSCC cohort, the cut-off values of serum OGN levels were determined by X-tile program, which were 12.651 ng/ml, (Fig. 1e). Patients were divided into 2 groups for further analysis (High serum level > 12.651 ng/ml, Low serum level ≤ 12.651 ng/ml). Although OGN level was not correlated with overall survival, but with regard to DFS, high OGN level was indicative of longer survivals (*P* = 0.032 Fig. 1f).

### OGN over-expression exhibits pleiotropic effects on colorectal cell lines in vitro

**Fig. 2:**
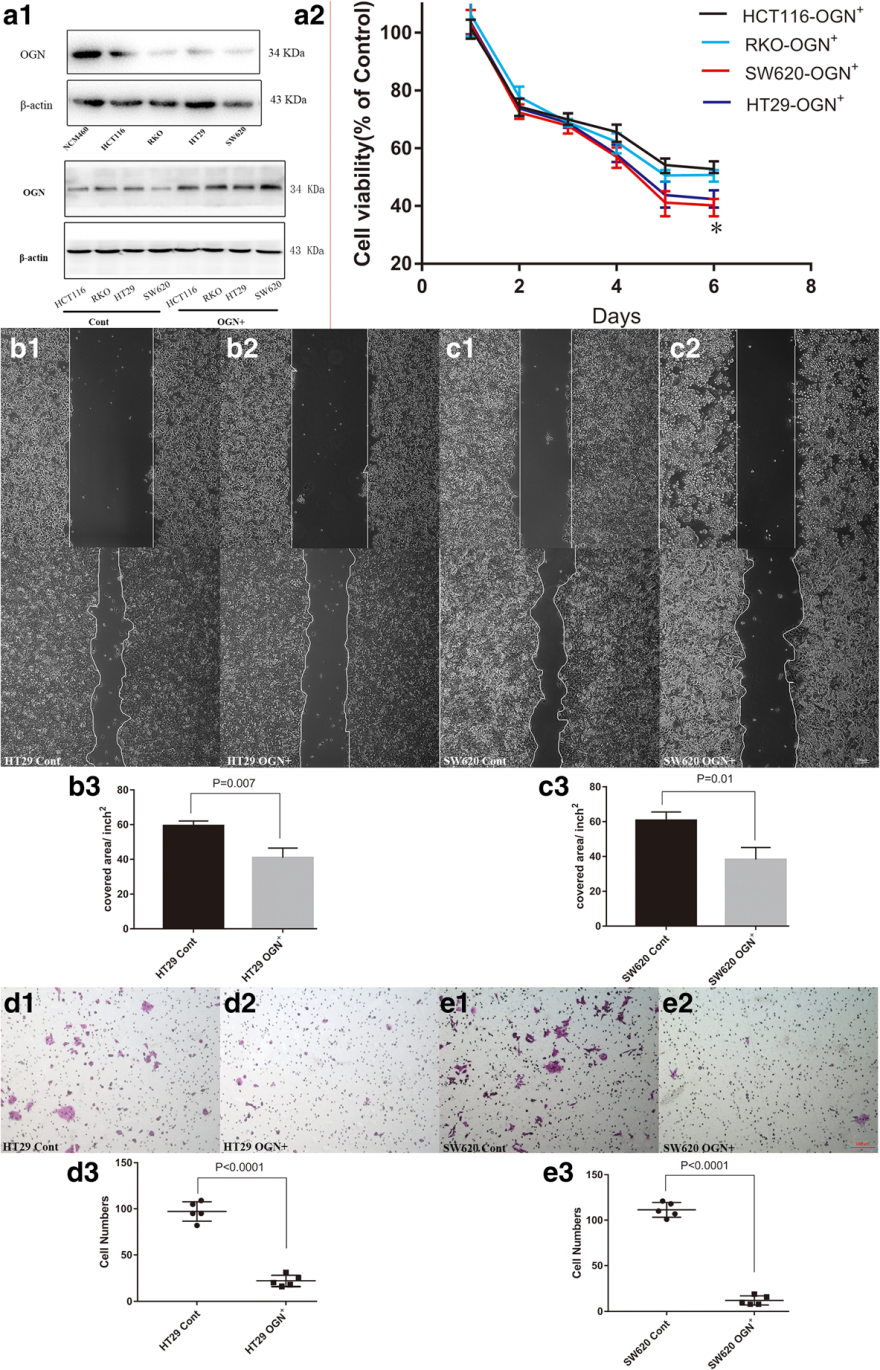
OGN was associated with viability and tumorigenic ability of colorectal cancer cells. ***a1***, The pattern of OGN in the normal human colon mucosal epithelial cells (NCM460) and colon cancer cell lines. Efficiency of OGN over-expression in cancer cell lines was measured by western blot. ***a2***, Influence of OGN expression on viability of cancer cells was measured by CCK-8 assay. * indicates *p* value less than 0.05 VS. control cells. Wound healing assay (**b**, **c**) and cell invasion (**d, e**). The results are expressed as the mean ± SD of three independent experiments

The expression of OGN in CRC cell lines were all at lower levels compared to the normal human colon mucosal epithelial cells (NCM460), see Fig. 2A1, so we created OGN over-expressing colorectal cell lines. The colon cancer cell lines were transduced with lentiviral constructs encoding human OGN cDNA to generate stable OGN over-expression cell lines (HCT116-OGN^+^, RKO-OGN^+^, SW620-OGN^+^, HT29-OGN^+^). Over-expression efficiency was verified by western blotting (Fig. 2A1). The CCK-8 assays were applied to examine whether OGN expression affected colorectal cancer cells proliferation and viability in vitro. As present in Fig. 2A2, there were significant growth suppression rates when OGN over-expressed in cell lines compared with the controls. What is more, cell lines SW620, HT29 showed better response to OGN challenging in cell CCK-8 assay. To determine the effect of altered OGN expression on migration and invasion, OGN transfected cells were used for wound-healing and transwell assays. Over-expression of OGN attenuated the migration and invasion ability of HT29 and SW620 cells (*p* < 0.05). In detail, OGN over-expression resulted in a 31.13% reduction in migration in the HT29 cells, and decreased the invasion by 37.01% in the SW620 cells (Fig. 2b, c). In line with the results obtained in the wound-healing assays, OGN over-expression prevented the average invasive cells of HT29 cells by 77.32%, as well as in SW620 cells, the average cells transwelled were reduced by 89.19% (Fig. 2d, e).

### OGN regulated the EGFR signaling pathway

**Fig. 3:**
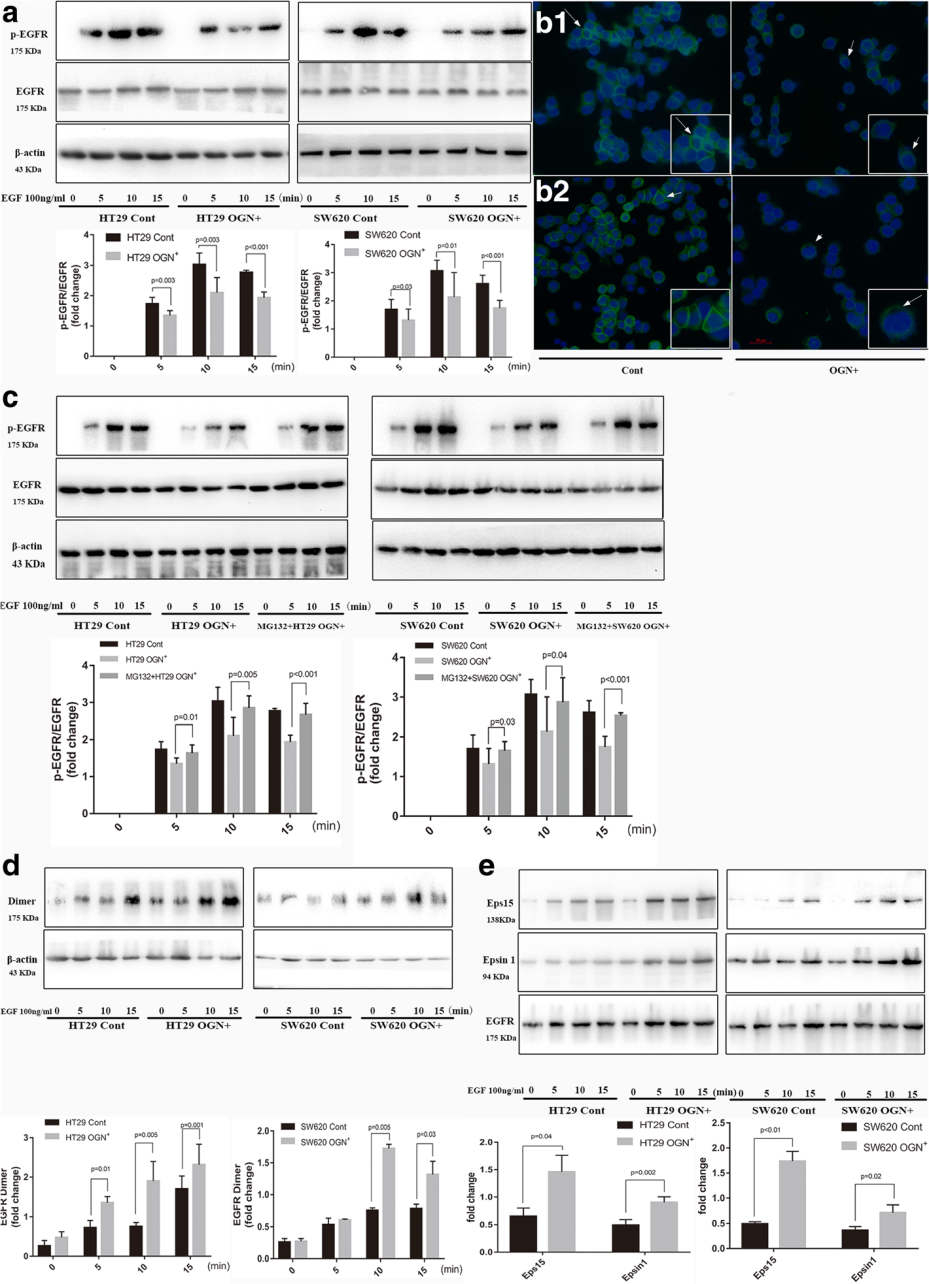
OGN increased dimerization, internalization, and degradation of EGFR. **a** HT29, SW620 cells with OGN over-expression were challenged with 100 ng/mL EGF for the indicated intervals. Cell lysates were subjected to Western blotting with indicated antibodies. **b** Immunofluorescent staining of cancer cells (B1: HT29, B2: SW620) with anti-EGFR antibody. Arrows show EGFR protein expression. **c** HT29, SW620 cells with OGN over-expression were treated with EGF (100 ng/mL) and co-cultured with or without MG132 (1 μM) for 24 h. **d** Cancer cells were treated with EGF (100 ng/mL) and then cultured with the cross-linker BS3 (3 mM) for 30 min at room temperature in order to demonstrate EGFR dimmers. **e** Recruitment of the endocytic adaptor Eps15 and epsin-1 is induced following OGN over-expression, when EGF stimulated for the indicated times

Pairwise gene correlation analysis for READ sets of TCGA was performed finding OGN correlated with EGFR closely (*p* = 0.0015). The growth of colorectal cancer cells is dependent on or facilitated by EGFR, hence, we explored whether EGFR and related signaling may be involved in OGN-induced cell inhibition. Firstly, we examined whether activation of the EGFR signaling pathway was modulated by OGN over-expression. HT29-OGN^+^ and SW620-OGN^+^ cells, which exposed to extracellular EGF (100 ng/ml), exhibited suppressed activation of EGFR protein phosphorylation level compared to Control cells. After 15 min of EGF exposure, the increase in p-EGFR/EGFR levels were reduced by 30.14% in HT29-OGN^+^ and 33.22% in SW620-OGN^+^ cells compared to the control cells (Fig. 3a). To address the mechanism for EGFR activation reduction, immunofluorescent staining of EGFR demonstrated a reduction in membranous, with a concomitant increase in cytoplasmic, after OGN over-expression (Fig. 3b). The signaling properties of EGFR are closely regulated by its sub-cellular localization, exactly, suppression of EGFR signaling may initiate from receptor endocytosis induction, eventually proteasome targeting and degradation of internalized receptors were followed. Hence, it was indicated that OGN enhanced the internalization of EGFR from the cell membrane into the cytoplasm, and then inhibited the activation of EGFR protein phosphorylation levels. Meanwhile we assessed whether OGN controlled EGFR activity by protein degradation via the proteasome. We observed the previously observed p-EGFR/EGFR inhibition was blocked, when HT29/SW620 cells with OGN over-expressed were pretreated with MG132 (the proteasome inhibitor) before exposing to EGF (Fig. 3c). The degradation was always resulted from the formation of EGFR dimmers, so we investigated whether OGN affected EGFR dimerization. Colon cancer cells were exposed to EGF for three-time intervals and then pretreated with the membrane-impermeable chemical cross-linker BS3 allowing resolution of dimeric components of EGFR. As a result, OGN over-expressed cells produced a greater increase in EGFR dimmers compared to the control cells (Fig. 3d). Besides the dimerization triggering EGFR endocytosis, the recruitment of EGFR internalization machinery, such as early endocytic adaptors (EGFR pathway substrate, Eps15 and epsin1) also played a role in the protein degradation. Therefore, we investigated whether the over-expression of OGN influenced the recruitment of Eps15 and epsin1 to EGFR. It was shown that the recruitment of Eps15 and epsin1 following EGF challenge was prominently increased in cells over-expressed for OGN (Fig. 3e). Collectively, these data implied a model in which over-expression of OGN detained proliferative signaling by inhibiting EGFR activity.

### OGN reduced Akt activity via inhibition of EGFR

**Fig. 4:**
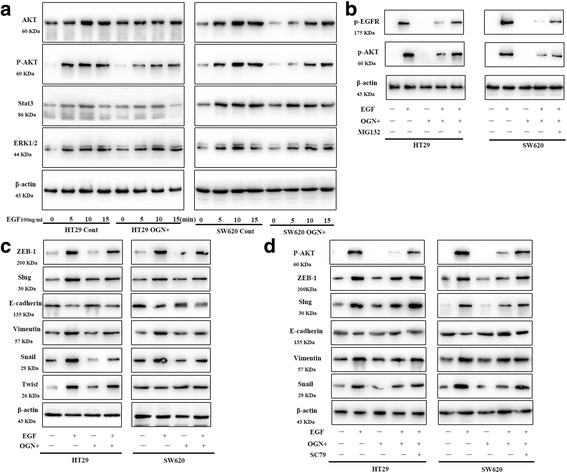
OGN reversed EMT through EGFR/Akt signal pathway. **a** HT29, SW620 cells with or without OGN over-expression were treated with EGF (100 ng/mL) for the indicated intervals. Cell lysates were subjected to Western blotting with the indicated antibodies referred to the three main downstream signals. **b** Cells with OGN over-expression were treated with EGF (100 ng/mL) and co-cultured with or without MG132 (1 μM) for 24 h. **c** Indicated cells were treated with EGF (100 ng/mL) for 15 min. Western blotting with the indicated antibodies regarding to EMT was performed. **d** Cells with OGN over-expression were challenged with EGF (100 ng/mL) and pretreated with SC79 (constitutive Akt activator) for 24 h. Western blotting with the significantly altered markers was performed

There are three major downstream pathways of EGFR, including PI3K/Akt, Stat3 and ERK1/2-mediated signaling pathways. Regarding to the observations above that OGN suppressed EGFR activity in HT29 and SW620 colorectal cancer cells, we aimed to identify which downstream pathways of EGFR will be inhibited after OGN over-expression. Firstly, the response of Akt, Stat3 and ERK1/2 to EGF exposure in OGN over-expressed cells were tested and only a decline in Akt phosphorylation was observed, while there was not any significant difference in Stat3 and ERK1/2 (Fig. 4a). As OGN abolished Akt activity, and then whether it was via EGFR that OGN reduced Akt activity? So, the proteasome inhibitor MG132, which referred to block EGFR phosphorylation, was used to pretreated cells with OGN over-expression, consequently the above observed Akt phosphorylation reduction was reversed as was the EGFR degradation blocked (Fig. 4b).

### OGN inhibited EMT via Akt activity

EGFR activation is frequently induced to mediate carcinoma invasion and metastasis through promoting EMT. Immunoblotting of protein lysates from OGN over-expressed HT29/SW620 cells abolished a significant reduction in expression of E-cadherin induced by EGF (Fig. 4c). Furthermore, the expressions of EMT-associated molecular biomarkers and EMT transcription factors, such as Slug, Zeb-1, and Snail decreased simultaneously, but had no effect of Twist (Fig. 4c). To assess whether suppression of p-Akt modulated the previously observed inhibition of EMT, the effect of OGN pretreated with SC79 (constitutive Akt activator) was explored again. Re-expression of p-Akt leaded to reverse the decrease in E-cadherin protein levels after OGN over-expression (Fig. 4d). Levels of EMT markers, such as Vimentin, Slug, Zeb-1, Snail and the protein levels of E-cadherin (Fig. 4d) were similarly affected, hence a critical role of Akt inhibition was indicated in the observed OGN-mediated decrease in EMT. The above results demonstrated the fact that OGN could reduce EMT via inhibition of the Akt pathway.

### Zeb-1 was essential for inhibition of EMT by OGN

**Fig. 5:**
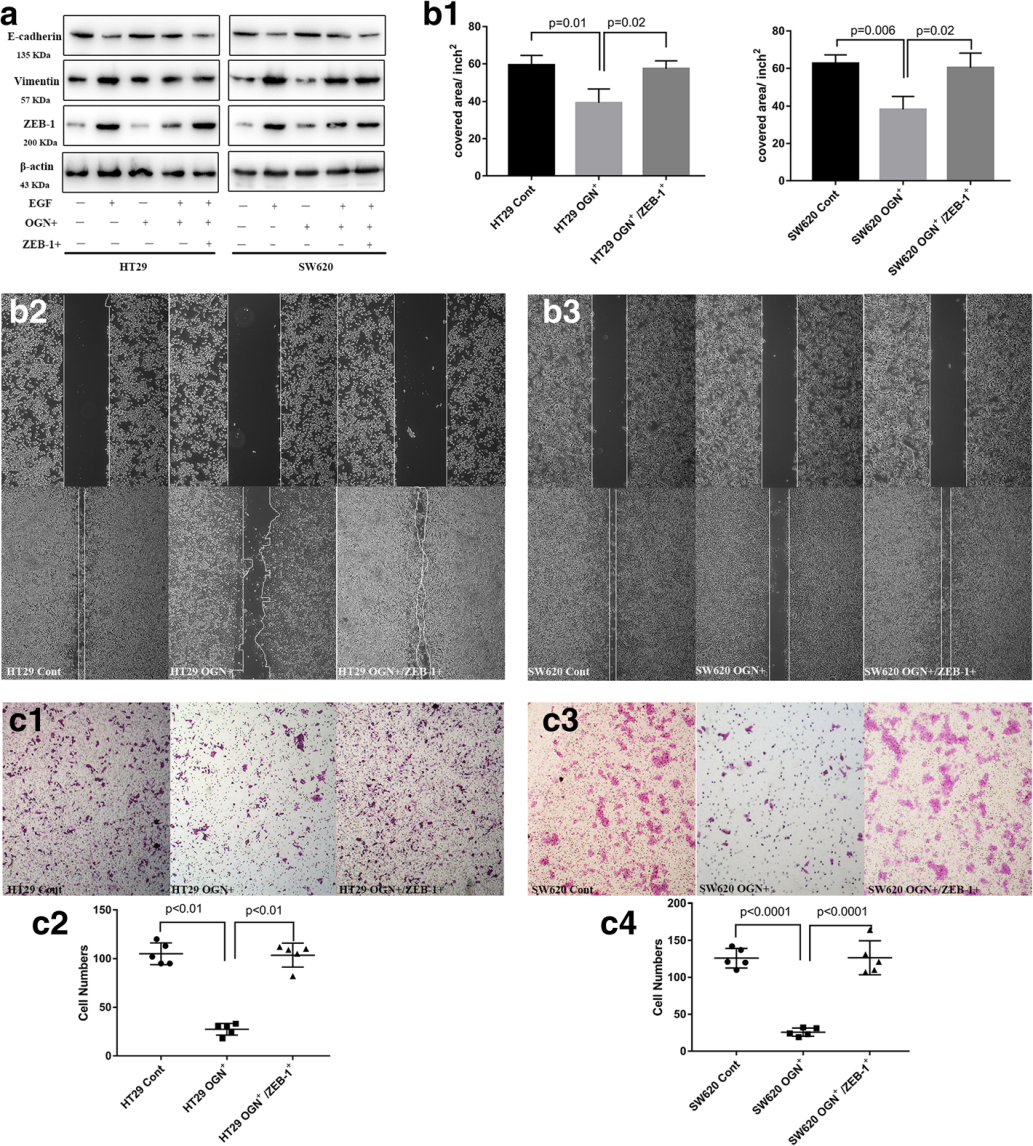
Zeb-1 was essential for inhibition of EMT by OGN. **a** Cells with OGN over-expression were challenged with EGF (100 ng/mL) and transiently transfected with Zeb-1. Western blotting with the significantly altered markers was performed. **b** Wound healing assay and (**c**), cell invasion assay. The results are expressed as the mean ± SD of three independent experiments

EMT plays a vital role in evolution of cancer cells gaining migratory and invasive properties. In order to confirm the ability of OGN to inhibit malignant potential and to determine the molecular mechanism by which OGN regulated EMT, we evaluated the altered EMT transcriptional markers above (Zeb-1, Slug and Snail) in OGN over-expressed cells and reassessed the cell invasion ability in rescue experiments. In rescue experiments, cancer cells with stable ectopic OGN expression were transiently transfected with Zeb-1, Slug or Snail. It has been confirmed that only rescue of Zeb-1 can reverse the inhibition of EMT in OGN over-expressed HT29/SW620 cells (Fig. 5a). wound-healing and transwell invasion assays were performed after treatment with ectopic OGN and EMT transcriptional markers expression. As shown in Fig. 5b–c, ectopic OGN expression markedly impeded the migration and invasion of HT29/SW620 cells, however, Zeb-1-over-expression abolished OGN-inhibition migration and invasion. These results indicated that Zeb-1 was a key molecular for OGN induced EMT inhibition and EMT signaling was essential for OGN-induced CRC cell migration and invasion.

### OGN consistently reduced tumorigenesis and metastasis in vivo

**Fig. 6:**
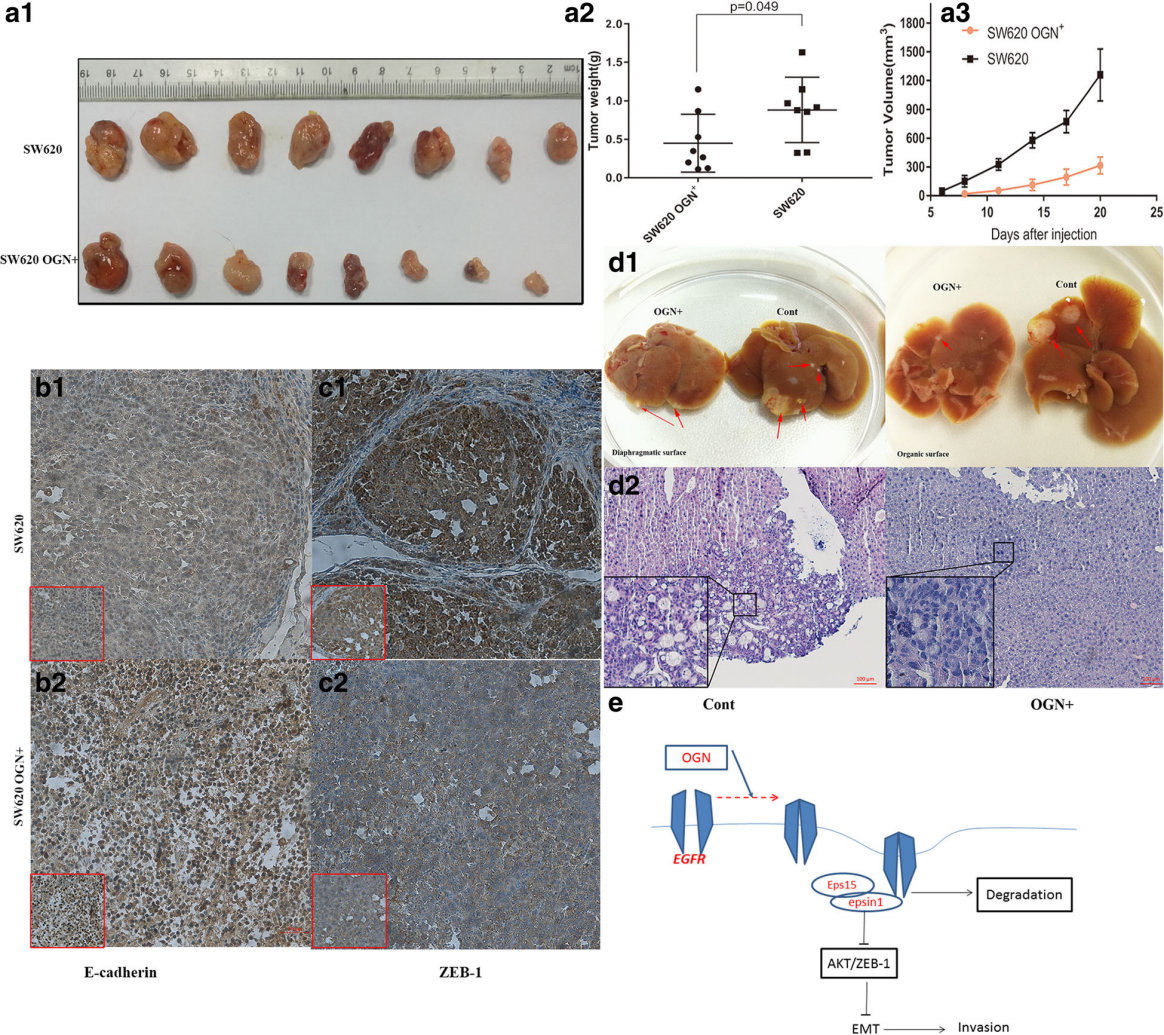
OGN consistently reduced tumor growth and liver metastasis in vivo. Equal numbers of OGN-over-expressing SW620 or their corresponding control counterparts were injected into nude mice. ***a1*** Representative photographs of tumor at the end of study. Tumors weight (***a2***) and tumor volumes (***a3***) were measured on the indicated days. IHC study showed strong E-cadherin (**b**), but weak Zeb-1(**c**) staining in OGN over-expressing tumors than its control group. ***d1*** The gross images of liver metastases observed in the nude mice injected with SW620 cells. Red arrows represent metastatic tumors. ***d2*** H&E staining for the liver metastases. **e** Schematic model depicts how OGN inhibits EMT through the EGFR/Akt signaling pathway

Understanding the effect of OGN in tumorigenicity requires consideration of the cancer cell per se and the microenvironment established by complex interactions between the host and the cancer cell. To validate our data in vitro models of colorectal cancer, we injected male nude mice subcutaneously with equal numbers of SW620-OGN^+^, or SW620 control cells and monitored both tumor growth and final tumor mass. Primary tumor growth and the emergence were assayed over time. OGN over-expression in cancer cells led to a decrease in growth rate and final mean tumor volume compared with control cells (Fig. 6a). In consistent with the above findings, as shown in Fig. 6c, xenograft tumors derived from OGN over-expressing cells had consistently less Zeb-1 staining compared to their control counterpart cells, at last, led to E-cadherin (marker of EMT) upregulation. In addition, the liver metastases were observed with fewer and smaller lesions in the mice injected with SW620-OGN^+^ (Fig. 6D1, D2). Collectively, these results suggested that OGN-reduced tumorigenicity and metastasis in vivo.

## Discussion

There are two main complementary findings highlighted in this study: 1) a positive association between OGN and improved survival in primary CRC tumors; and 2) reduced activation of EGFR/AKT/Zeb-1 in colorectal cancer cells when OGN over-expressed. A new underlying mechanism is indicated here shown in Fig. 6e, in which OGN impeded EGFR kinase activation and attenuated the downstream activators Akt and Zeb-1 via affecting EGFR dimerization, internalization and recruitment of Eps15 and epsin1 to EGFR. Moreover, reduced Zeb-1 inhibited EMT of cancer cells per se and decreased tumorigenesis. Together, these data offer a unifying mechanism for the clinical observations linking high levels of OGN with reduced relapse and death in patients with CRC.

To our knowledge, this study is the first to describe OGN expression in a cohort of colorectal cancer patients in which protein expression and serum levels are associated with a longer survival. To explain this, a specific chromosomal organization of OGN has been suggested in this study and others. For instance, a p53 DNA-binding sequence in OGN was identified and confirmed, resulting in the activation of the OGN gene. Since, the known tumor suppressor p53 was supposed to be in charge of OGN gene expression [18]. Coincidentally, p53 is frequently mutated to inactivated in many different tumors, including colorectal cancers, breast, lung, ovary and prostate cancers, in which a reduction or absence of OGN expression was observed [6]. So, it was not a surprise that OGN expression was markedly associated with longer survival time, with prolonged cancer specific survival as 75.7 months in the High OGN expression group versus 61.6 months in the Low OGN expression group. In addition, the OGN promoter contains three conserved AP-1-binding sites [19], prompting the validation of OGN as a target gene downstream to LIP/MAPK/AP-1 [20]. Then cell death can be induced by activating MAPK/AP-1 and OGN expression. Hence, it was observed that ER stress-triggered cell death was attenuated by specific knockdown of OGN mRNA in melanoma cells. Clones transfected with OGN were significantly more sensitive to ER stress-triggered cell death following ER inducer. Consistently, in our study the high serum OGN level was also indicative of longer survival with regard to DFS in CRC. Hence, we speculate that the role of OGN in cell inhibition is rather general and not cell type specific.

Despite numerous studies demonstrating altered OGN expression in cancers, functional data on how OGN involved in cancer pathology are lacking, and further research is needed. That is why this study was carried out for the underlying mechanism, and we observed OGN can reduce Akt activity via inhibition of EGFR. In detail, OGN increased dimerization of EGFR, then triggered EGFR endocytosis and induced the recruitment of downstream components of the EGFR internalization machinery, such as early endocytic adaptors (EGFR pathway substrate, Eps15 and epsin1). In agreement with these findings, OGN was previously indicated to inhibit not only vascular smooth muscle cells proliferation [21], but also keratocyte [22] and fibroblasts proliferation [23]. This effect has been illustrated to associate with various cytokines just like: bFGF, TGF-β, platelet-derived growth factor (PDGF), and angiotensin II (Ang II) [24, 25]. In accordance, the malignant lesion can secrete various factors such as bFGF, TGF-β, PDGF, EGF creating tumor microenvironment by interacting through other growth factors receptors (EGFR) [26]. Furthermore, our work unambiguously identified the OGN/EGFR/Akt signaling pathway as a mechanism by which OGN inhibited CRC cell survival and proliferation. These results were further validated by related histopathologic observations in carefully annotated human CRC samples and xenograft tumors. Together, these observations provided strong evidence that OGN is a critical host modifier of CRC cell growth and survival, since EGFR plays a pivotal role in progression of CRC [27].

Above all, our experimental approach has yielded novel findings that OGN can affect EMT, a vital mechanism involved in tumor development and progression [28, 29]. Our results indicated that OGN induced Zeb-1 reduction, eventually reversion of EMT. Besides the mechanism mentioned above, in further studies we need evaluate which transcriptional factor would be the direct downstream of OGN. HIF-1α may be one of the candidates, as OGN was correlated with cell metabolism based on a variety of clues implicated in here and others. First of all, OGN expression also has been observed to associate with the metabolism, as altered OGN protein was found in obese humans [30]. Furthermore, OGN has been identified as a quantitative trait loci (QLT), relevant to the metabolic syndrome on chromosome 17 [31, 32]. In addition, a recent study showed that OGN was demonstrated to highly expressed and secreted into the circulation in mice, and functions as an adipose hormone to limit food intake, possibly by increasing IL-1b and IL-6 [33]. In coincidence, HIF-1α plays a key role in reprogramming cell metabolism from oxidative phosphorylation to aerobic glycolysis, in which the malignant behavior is triggered [34, 35]. Meanwhile, HIF-1α is frequently induced through EGFR activation and mediates carcinoma angiogenesis as well as promotes EMT and metastasis.

Collectively, our work identifies decreased OGN expression in CRC impaired EGFR internalization as a novel mechanism through which tumor cells induce EGFR activity to sustain proliferative signaling. Our findings of OGN-dependent regulation of EGFR internalization add alternative mechanism to a growing body of evidence that highlights the importance of receptor endocytosis in cancer progression. Thus, our findings highlight the importance of the OGN-mediated of EGFR signaling in tumor biology, revealing a novel mechanism regulating CRC growth and progression.
