# Correction: Osteoglycin (OGN) reverses epithelial to mesenchymal transition and invasiveness in colorectal cancer via EGFR/Akt pathway

**DOI:** 10.1186/s13046-026-03704-8

**Published:** 2026-04-10

**Authors:** Xiang Hu, Ya-Qi Li, Qing-Guo Li, Yan-Lei Ma, Jun-Jie Peng, San-Jun Cai

**Affiliations:** 1 Department of Colorectal Surgery, Fudan University Shanghai Cancer Center, 270 Dong’an Road, Shanghai, 20032 China; 2 Department of Oncology, Shanghai Medical College, Fudan University, Shanghai, 200032 China

**Correction: J Exp Clin Cancer Res 37, 41 (2018)**

**https://doi.org/10.1186/s13046-018-0718-2**

Following publication of the original article [1], the authors found errors in Fig. 5c. Figure 5C1 HT29 Cont and Figure 5 C3 SW620 Cont should show the image of HT29 and SW620 cells, respectively. The correct figure is given below:

Incorrect Fig. 5

**Fig. 5:**
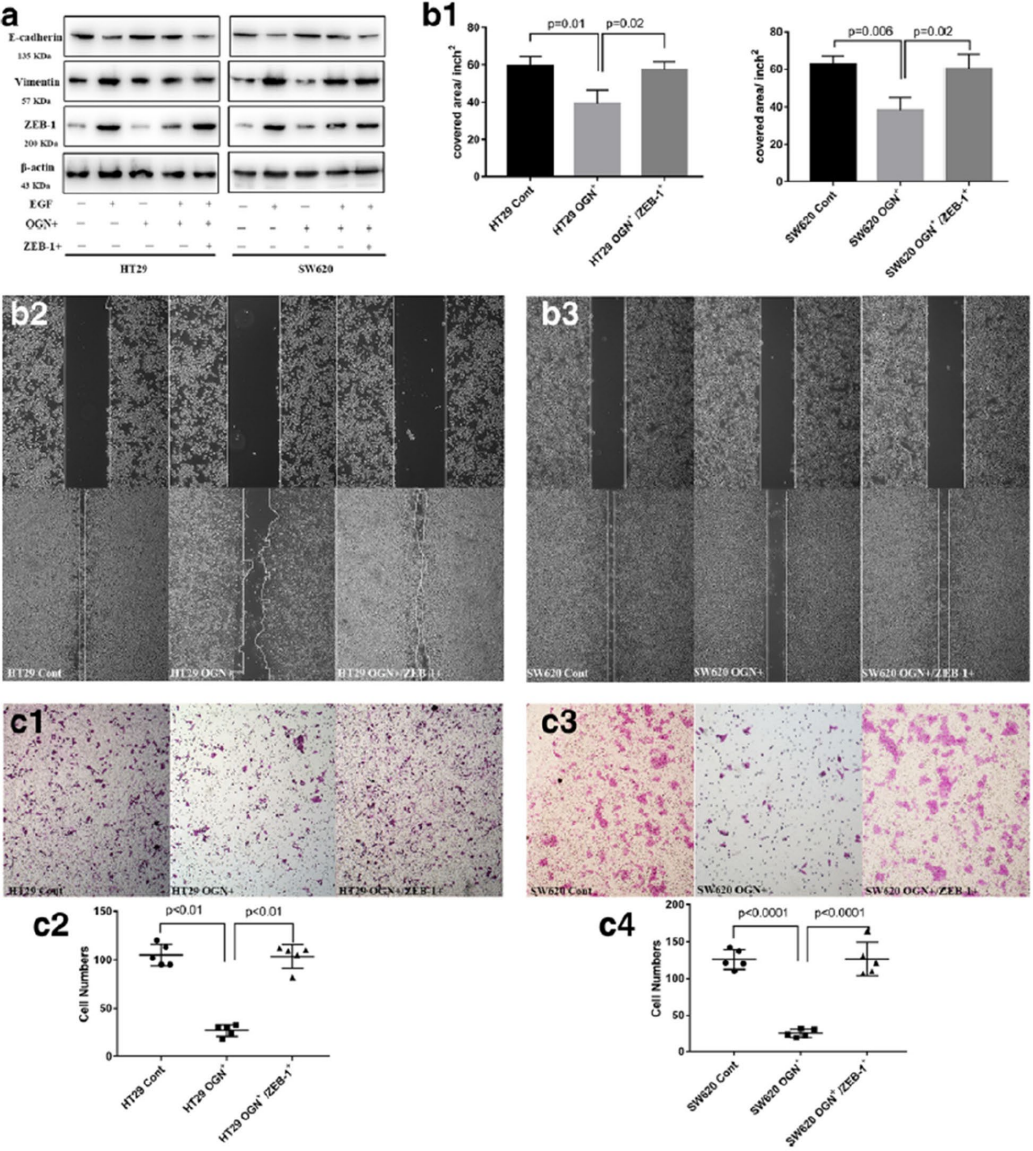
Zeb-1 was essential for inhibition of EMT by OGN. **a** Cells with OGN over-expression were challenged with EGF (100 ng/mL) and transiently transfected with Zeb-1. Western blotting with the significantly altered markers was performed. **b** Wound healing assay and (**c**), cell invasion assay. The results are expressed as the mean ± SD of three independent experiments

Correct Fig. 5

**Fig. 5:**
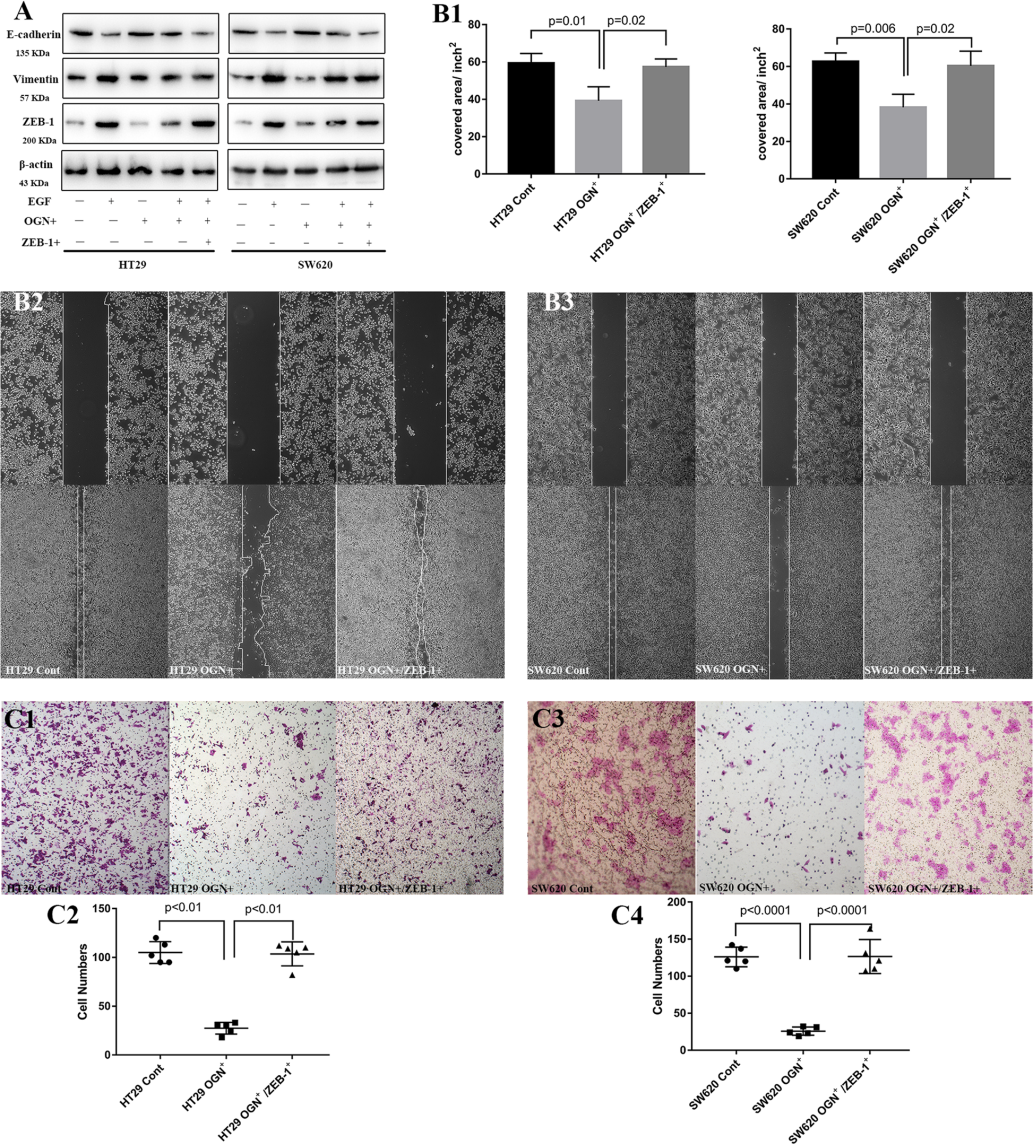
Zeb-1 was essential for inhibition of EMT by OGN. **a** Cells with OGN over-expression were challenged with EGF (100 ng/mL) and transiently transfected with Zeb-1. Western blotting with the significantly altered markers was performed. **b** Wound healing assay and (**c**), cell invasion assay. The results are expressed as the mean ± SD of three independent experiments

## References

[1] Hu X, Li YQ, Li QG, et al. Osteoglycin (OGN) reverses epithelial to mesenchymal transition and invasiveness in colorectal cancer via EGFR/Akt pathway. J Exp Clin Cancer Res. 2018;37:41. 10.1186/s13046-018-0718-2. 29499765 10.1186/s13046-018-0718-2 PMC5833032

